# False-Positive Urine Ethyl Glucuronide Results in Pregnancy Caused by *Escherichia coli* Urinary Tract Infection

**DOI:** 10.1097/og9.0000000000000195

**Published:** 2026-08-06

**Authors:** Jasmin E. Charles, Lillian Hayes, Michael Moss, Marcela C. Smid

**Affiliations:** Department of Obstetrics and Gynecology, Department of Family Medicine, and Department of Emergency Medicine, University of Utah Health, Salt Lake City, Utah; and Community Health Clinics, Salt Lake City, Utah.

## Abstract

**BACKGROUND:**

Urine alcohol biomarker testing is commonly used to monitor alcohol use during pregnancy but may be difficult to interpret when results are discordant or influenced by physiologic or microbiologic factors.

**CASE:**

We describe a 27-year-old pregnant woman with opioid and methamphetamine use disorder, atypical preeclampsia, and recurrent *Escherichia coli* urinary tract infection who denied alcohol use. Repeated urine testing showed positive urine ethyl glucuronide with negative ethyl sulfate and ethanol. Whole-blood phosphatidylethanol testing confirmed no systemic alcohol exposure. Findings were consistent with postcollection bacterial synthesis of ethyl glucuronide in bacteriuric urine specimens.

**CONCLUSION:**

This case highlights the need for careful interpretation of alcohol biomarkers in perinatal addiction care because false-positive results may affect treatment decisions and child protective service involvement.

## Abstract 2

Accurate interpretation of alcohol biomarkers in pregnancy is critical because results may affect clinical care and child custody decisions.

**Teaching Points**

- Urine ethyl glucuronide–positive, ethyl sulfate–negative alcohol biomarker results in the setting of bacteriuria should raise suspicion for postcollection bacterial ethyl glucuronide synthesis rather than alcohol consumption.
- Discordant urine alcohol biomarkers should prompt confirmatory testing with phosphatidylethanol and careful review of specimen collection and handling.
- Accurate interpretation of alcohol biomarkers in pregnancy is essential because false-positive results may affect treatment decisions, child welfare involvement, and legal outcomes.

Urine ethyl glucuronide and ethyl sulfate are sensitive and widely used biomarkers for detecting recent alcohol use, remaining detectable for 1–4 days after alcohol consumption.^1^ Phosphatidylethanol levels can be used for confirmatory testing and may remain detectable for 3–5 weeks after alcohol exposure.^2^ In perinatal and addiction medicine settings, pregnant women are frequently screened with urine toxicology testing for alcohol and other substances. However, false-positive ethyl glucuronide results can occur as a result of bacterial fermentation of glucose to ethanol and subsequent glucuronidation in stored bacteriuric urine specimens. The Substance Abuse and Mental Health Services Administration has highlighted this risk in an advisory addressing interpretation of alcohol biomarkers in toxicology testing.^3^ *Escherichia coli* and other enteric bacteria have been shown to produce ethanol and ethyl glucuronide in vitro, whereas ethyl sulfate is not formed under these conditions.^4^ Awareness of this mechanism is critical to prevent misinterpretation of laboratory data, particularly among pregnant women in recovery programs for whom these results may influence may affect clinical care, legal reporting, and child welfare decisions.

## CASE

A 27-year-old G3P2002 at 36 1/7 weeks of gestation presented for her first visit at a multidisciplinary perinatal addiction clinic affiliated with a residential treatment program for pregnant and parenting women with substance use disorders. She received prenatal care with a community clinician before entering residential treatment. Her medical history included opioid (fentanyl) and methamphetamine use disorders, nicotine use disorder, recurrent urinary tract infections, depression, anxiety, and attention-deficit/hyperactivity disorder. She was taking buprenorphine/naloxone 16 mg/4 mg daily and reported last fentanyl and methamphetamine use approximately 1 month before the initial presentation. Nicotine lozenges were prescribed for smoking cessation, and sertraline 25 mg daily and hydroxyzine 25 mg as needed were initiated for mood and anxiety symptoms. She reported a prepregnancy history of occasional alcohol use but no use during pregnancy. She denied consuming alcohol-containing substances (eg, vanilla abstract) or excessive use of alcohol-based hand disinfectant.

At the intake visit, she was afebrile and normotensive (110/70 mm Hg) and reported dysuria. Urine culture was positive for *E coli* above 100,000 colony-forming units/mL, resistant to ampicillin and ciprofloxacin, and intermediate to ampicillin–sulbactam (Table 1). Laboratory results showed aspartate transferase 114 units/L, alanine transaminase 166 units/L, platelets 351×10^9^/L, normal bilirubin, and negative viral hepatitis and syphilis serologies. Concern for evolving atypical preeclampsia prompted induction of labor at 37 1/7 weeks of gestation. She had an uncomplicated vaginal birth of a healthy term female infant. Liver enzymes normalized within 72 hours postpartum. She was discharged with the infant to the residential treatment program.

**Table 1.** Urine Culture and Toxicology Results

GA	Key Results	Context and Interpretation
32 1/7 wk	Urine culture more than 100,000 CFU/mL *E coli*	*E coli* UTI; antibiotics given
36 1/7 wk	Urine culture more than 100,000/mL CFU *E c*oli	*E coli* UTI; antibiotics given
36 4/7 wk	Urine EtOH not detected; EtG more than 4,000 ng/mL, EtS not detected	Discordant biomarkers (EtG+/EtS−) despite no reported alcohol use
37 1/7 wk	Vaginal delivery for preeclampsia with severe features (elevated LFTs, proteinuria)	Not applicable
PPD 7	Urine EtOH, EtG, and EtS not detected	Negative (EtG-/EtS−)
PPD 9	EtG 1,138 ng/mL; EtS not detected	Recurrent EtG-positive/EtS-negative discordance
PPD 13	EtG 2,025 ng/mL; EtS not detected	Recurrent EtG-positive/EtS-negative discordance
PPD 21	EtG 1,154 ng/mL; EtS not detected	Recurrent EtG-positive/EtS-negative discordance
PPD 30	Urine culture>100,000 CFU/mL *E coli*	Recurrent *E coli* UTI
PPD 31	Whole blood PEth less than 10 ng/mL	No systemic ethanol exposure

CFU, colony forming units; UTI, urinary tract infection; GA, gestational age; *E coli*, *Escherichia coli*; EtOH, ethanol; EtG, ethyl glucuronide; EtS, ethyl sulfate; LFT, liver function test; PPD, postpartum day; PEth, phosphatidylethanol.

Results of buprenorphine metabolites not shown as consistent with reported use.

The residential treatment program was concerned about recurrent positive alcohol metabolites in their routine toxicology testing. Additional interpretation and evaluation were requested given concerns of child protective services. Urine ethyl glucuronide and ethyl sulfate were tested with initial immunoassay screening followed by quantitation through liquid chromatography–tandem mass spectrometry. Whole-blood phosphatidylethanol was determined by liquid chromatograph–tandem mass spectrometry. We reviewed all available toxicology testing, which are summarized in Table 1. Negative ethyl sulfate and ethanol across all specimens, combined with repeated *E coli* bacteriuria and negative phosphatidylethanol, supported postcollection bacterial formation of ethyl glucuronide rather than recent alcohol consumption. All urine samples tested negative for oxidants or adulteration. We reviewed results with the patient, and she provided written consent for publication of this case report. A report documenting our interpretation was provided to the residential treatment program, child protective services caseworker, and presiding judge.

## DISCUSSION

This case highlights a critical pitfall in interpreting urine alcohol biomarkers with important clinical and psychosocial implications for pregnant women with substance use disorder. As in the case of our patient, ethyl glucuronide–positive, ethyl sulfate–negative, and ethanol-negative results in the setting of urinary tract infection may represent false-positive findings caused by bacterial metabolism rather than alcohol consumption. *E coli* and other enteric bacteria may contribute to postcollection ethyl glucuronide formation through several mechanisms. Bacteria and yeast, including *Candida albicans*, can ferment urinary glucose to produce ethanol, particularly in the presence of glucosuria. In the presence of ethanol, bacterial metabolism may subsequently facilitate in vitro glucuronidation, leading to detectable ethyl glucuronide concentrations even without alcohol ingestion.

The underlying mechanism has been characterized in vitro. Helander et al^4^ demonstrated that urine contaminated with *E coli*, *Klebsiella pneumoniae*, or *Enterobacter aerogenes* can undergo in vitro ethanol production and ethyl glucuronide synthesis when stored at room temperature. Under identical conditions, ethyl sulfate failed to form because its formation requires hepatic enzymatic sulfation and cannot occur through bacterial processes. Thus, ethyl glucuronide–positive/ethyl sulfate–negative patterns in ethanol-negative specimens suggest postcollection artifact. This may be particularly important in pregnancy when recurrent urinary tract infections may be more frequent.

Clinical laboratory guidance further cautions that discordant ethyl glucuronide and ethyl sulfate results should not be used alone to confirm recent alcohol exposure because they may reflect bacterial contamination, delayed refrigeration, or specimen handling issues.^3,4^ Use of preservatives such as sodium fluoride and prompt specimen refrigeration may reduce postcollection bacterial metabolism and improve interpretation of urine alcohol biomarkers. In addition, confirmatory phosphatidylethanol testing, reflecting in vivo ethanol metabolism, may be helpful when reported alcohol use is discordant with urine biomarker results. Although phosphatidylethanol cannot exclude minimal alcohol exposure, consumption of approximately 1.5–2 drinks daily generally produces concentrations more than 20 ng/mL in women.^5^ Urine ethyl glucuronide concentrations greater than 1,000 ng/mL are more commonly associated with heavy alcohol consumption within 1–2 days or light drinking on the same day.^3,6^

False-positive alcohol biomarker results carry significant clinical and psychosocial implications. For pregnant and postpartum women in recovery, these interpretive nuances are especially important. Misclassification of alcohol exposure based on false-positive ethyl glucuronide results may have serious psychosocial consequences, including stigma, treatment disruption, or unwarranted child protective service referrals.^7,8^ Confirmatory phosphatidylethanol testing and collaboration among obstetric, addiction medicine, and laboratory teams are recommended to support accurate interpretation and patient-centered care. Careful interpretation of discordant alcohol biomarkers is particularly important in perinatal addiction care, where toxicology results may directly influence treatment access, custody determinations, and legal outcomes.

## References

[1] Walsham NE Sherwood RA. Ethyl glucuronide. Ann Clin Biochem 2012;49(pt 2):110–7. doi: 10.1258/acb.2011.011115 22113954

[2] Haber PS. Identification and treatment of alcohol use disorder. N Engl J Med 2025;392:258–66. doi: 10.1056/NEJMra2306511 39813644

[3] Substance Abuse and Mental Health Services Administration. The role of biomarkers in the treatment of alcohol use disorders, 2012 revisions, advisory, volume 11, issue 2. Accessed May 11, 2026. https://www.niaaa.nih.gov/sites/default/files/SAMHSA-Advisory-Role-of-Biomarkers-in-the-Treatment-of-Alcohol-Use-Disorder-2012.pdf

[4] Helander A Olsson I Dahl H. Postcollection synthesis of ethyl glucuronide by bacteria in urine may cause false identification of alcohol consumption. Accessed May 11, 2026. https://scispace.com/pdf/postcollection-synthesis-of-ethyl-glucuronide-by-bacteria-in-5bcar0adi1.pdf 10.1373/clinchem.2007.089482 17717128

[5] Ulwelling W Smith K. The PEth blood test in the security environment: what it is; why it is important; and interpretative guidelines. J Forensic Sci 2018;63:1634–40. doi: 10.1111/1556-4029.13874 30005144

[6] Jatlow P O'Malley SS. Clinical (nonforensic) application of ethyl glucuronide measurement: are we ready? Alcohol Clin Exp Res 2010;34:968–75. doi: 10.1111/j.1530-0277.2010.01171.x PMC3711108 20374218

[7] Kurtz T Charron E Shakib J Smid MC. Drug testing interpretation in the peripartum setting: results of clinician survey. J Addict Med 2024;18:595–8. doi: 10.1097/adm.0000000000001322 38842171

[8] Kurtz T Smid MC. Challenges in perinatal drug testing. Obstet Gynecol 2022;140:163–6. doi: 10.1097/aog.0000000000004808 35852264

